# Sleep and hypertension

**DOI:** 10.1007/s11325-019-01907-2

**Published:** 2019-08-12

**Authors:** B. Han, W. Z. Chen, Y. C. Li, J. Chen, Z. Q. Zeng

**Affiliations:** 1 Department of Preventive Medicine, Chengdu Medical College, Chengdu, China; 2 Department of Epidemiology and Statistics, Chengdu Medical College, Chengdu, China; 3 School of Public Health, Chengdu Medical College, Chengdu, China

**Keywords:** Sleep, Hypertension, Obstructive sleep apnea, Snoring

## Abstract

**Purpose:**

Hypertension is a global public issue, and sleep status was regarded as its risk factor; however, the results were inconsistent. This study aims to deeply investigate the correlation between sleep status and hypertension.

**Methods:**

The electronic databases Cochrane Library, Pubmed, and Embase updated to May 31, 2019, were retrieved. Studies were selected according to the predefined screening criteria, and their qualities were assessed by using quality check scales. Based on Stata 15.1 software, the associations between sleep status and hypertension were analyzed by meta-analyses, using odds ratio and 95% confidence interval as effect indexes. Furthermore, publication bias and small study bias were evaluated using Begg and Egger’s test. In addition, sensitivity analysis was conducted through ignoring one study per time and then observing its influences on the pooled results.

**Results:**

A total of 54 studies (involving 1,074,207 subjects) were eligible for this meta-analysis. Six factors were included in this study. Raised blood pressure was associated with obstructive sleep apnea (OSA), oxygen desaturation index (ODI), short sleep duration, and long sleep duration. The differences in ≤ 5 h, 6 h, ≥ 9 h, and 10 h groups had statistical significances, while there was no significant difference in ≥ 8 h group. Snoring is a risk factor of hypertension (OR = 1.94, 95%CI 1.41–2.67). Subgroup analysis was conducted and results were varied.

**Conclusions:**

The hypertension risk might be reduced by treated OSA, ODI, and snoring, as well as appropriate sleep duration. More studies with large sample sizes and high qualities should be included to support the findings further.

**Electronic supplementary material:**

The online version of this article (10.1007/s11325-019-01907-2) contains supplementary material, which is available to authorized users.

## Introduction

Hypertension, also known as high or raised blood pressure, is a condition in which the blood vessels have persistently raised pressure [1]. It is a global public health issue, as well as an important factor for cardiovascular diseases [2–4]. It has been reported that one in four men and one in five women have raised blood pressure. Previous studies reported that hypertension was caused by both genetic and environmental factors. Some lifestyle factors, such as smoking, excess salt, drinking, and unhealthy sleep duration, have an effect on the incidence of hypertension [5].

Sleep status alters autonomic nervous system function and other physiologic events that influence blood pressure. In addition, unhealthy sleep status can change the blood pressure response and increase hypertension risk [6]. Habitual sleep duration shorter than the median of 7–8 h is related to the evaluated incidence of hypertension, which is more common for people sleeping for less than 6 h per night [7]. Some studies reported that increased sleep time also was a risk factor for hypertension [8]. The association between obstructive sleep apnea (OSA) and hypertension was commonly reported, about 50% patients with OSA are hypertensive, and an estimated 30 to 40% of patients with hypertension have OSA [9].

Even though a vast number of studies agreed with that sleep conditions were associated with hypertension, the results of this relationship were not completely consistent in different population. For instance, short sleep duration was found to be a risk factor in American population [8], while the significant association was not reported in Chinese population [10]. In terms of sleep apnea, positive association (OR = 1.72, AHI ≥ 30) was found in Malaysian young adults [11], there is, however, no significant results were found in French population [12]. Recent data on the effects of sleep status on hypertension should be explored. There are numerous meta-analyses done on relationship between sleep and hypertension, while the evidence still lacks for comprehensive summary for multiple subtypes of sleep status. Then, to prevent the bias caused by different researches, we performed meta-analyses for sleep status with sufficient available datasets to find cumulative epidemiological evidence for the significant effects contributed dependently or independently by varied subtypes of sleep status to hypertension. This study might lay a theoretical foundation for preventing hypertension.

## Methods

### Search strategy and selection criteria

We systematically searched PubMed, Embase, and Web of Science to identify the association studies (cross-sectional studies, case-control studies, and cohort studies) published before May 31, 2019, using key terms “blood pressure or hypertension or BP or systolic pressure or diastolic pressure or SBP or DBP” and “sleep disorder or sleep duration or sleep apnea or snoring or excessive daytime sleepiness or sleep quality.” We also screened references of all included studies, reviews, meta-analyses, pooled analyses, and related citations in PubMed to identify additional publications.

### Inclusion and exclusion criteria

Studies satisfied below criteria were eligible: (1) were published in a peer-reviewed journal in English; (2) reported the associations of sleep status and hypertension; (3) provided risk estimates (e.g., risk ratios (RRs), odd ratios (ORs), 95% confidence intervals (CIs), regression coefficients and standard errors) or sufficient data to calculate these estimates.

The exclusion criteria were as follows: (1) lacked sufficient information; (2) were not published as full research articles, such as reviews, comments, or letters; (3) were studies on treatment and disease mortality or survival (rather than incidence). Two investigators (Z.ZQ and H.B) assessed the eligibility of each study independently.

### Data extraction

Data extraction was completed by two investigators and subsequently checked by two others. Data collected using the predesigned standard forms, including first author, publishing year, PubMed identifier number, study design, method of case selection, source population, ethnicity of participants, sample size, and risk estimates. When more than one publication used same study population, we collected data from the most recent or the largest one. The references regarding cohort study and case-control study were assessed using Newcastle Ottawa assessment scale, articles focused on cross-sectional study were evaluated using adjusted Newcastle Ottawa assessment scale (see supplementary 4).

### Statistical analysis

We combined the risk estimates of factors from each of the included studies to obtain summary statistics of associations. The RRs or ORs and their 95% CIs were considered effect indices. Meta-analyses were conducted only for factors with at least three independent datasets. Subgroup analyses were conducted according to ethnicity or study design if the number of datasets was more than five. For studies reporting the categorical results, the risk estimates of factors were calculated using the generalized least squares method by assuming linearity of the natural log-scale ORs. Cochran’s *Q* statistic and *I*^2^ statistic are used to quantify heterogeneity for the included studies [13, 14]. For high heterogeneity outcome (*I*^*2*^ > 50%), the random-effects model was utilized for obtaining the pooled results. For homogeneous outcomes, such as low and moderate heterogeneity (*I*^*2*^ ≤ 50%), the fixed-effects model was applied to pool the effect indexes. Publication bias of the included studies was evaluated using Begg’s test [15]. Potential small study bias was assessed by Egger’s test [16]. For sensitivity analysis, one study was ignored at a time and its influences on the pooled results were observed. Statistical analyses were conducted using Stata 15.1 software (https://www.stata.com/).

## Results

### Characteristics of eligible studies

**Fig. 1:**
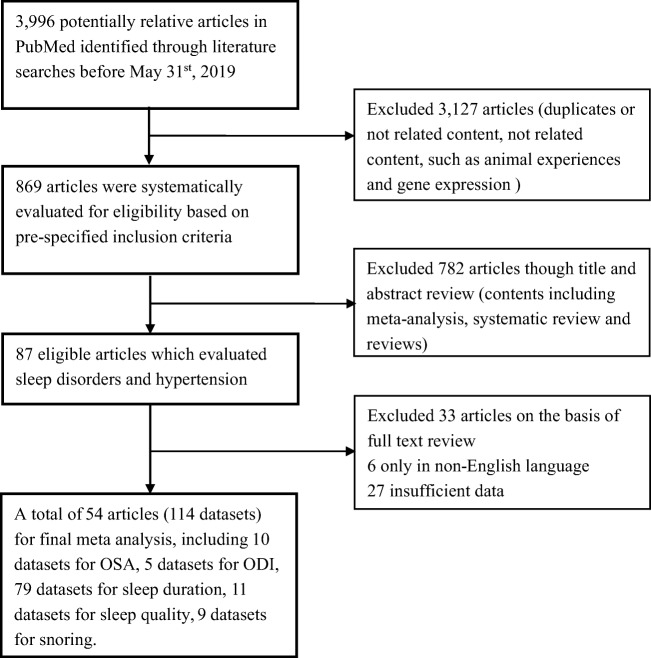
Literature search results

Total 54 publications including 1,074,207 subjects were included in our study (Fig.1). We excluded 3127 irrelevant and duplicate publications. We further excluded 782 articles after title or abstract review, and another 33 articles were not included in our meta-analyses due to non-English languages or insufficient data. Finally, 54 articles (see supplementary 1) including a total of 114 datasets were selected for associations between sleep status and hypertension (see supplementary 2). Among those datasets, 10 datasets including 13,274 subjects were for associations between OSA and hypertension, five datasets including 5,334 subjects were for associations between oxygen desaturation index (ODI) and hypertension, 79 datasets involving 949,489 subjects for association between sleep duration and hypertension, 11 datasets involving 53,897 subjects for association between sleep quality and hypertension, and 5 datasets involving 90,247 subjects for association between snoring and hypertension.

### Obstructive sleep apnea and hypertension

A total of 10 articles were included in the meta-analysis, including two cohort studies, one case-control study, and seven cross-sectional studies. A summary of finding regarding association between OSA and hypertension was shown in Table 1. A significant association between OSA and hypertension was found (OR = 1.798, 95%CI 1.355–2.384). Furthermore, we conducted subgroup analysis to evaluate the associations in different study design groups. In cross-sectional study group, the combined results showed OSA was correlated with hypertension significantly (OR = 1.980, 95%CI 1.312–2.987).

**Table 1:** Summarized risk estimate for the association between sleep disorders and hypertension

Factor	Group	Datasets	OR	95%CI		*p* value	*I*^2^	*p* value
				Lower	Upper			
OSA	Total	10	*1.798*	*1.355*	*2.384*	*4.70 × 10*^*−5*^	72.4%	0.000
	Asian	4	*1.998*	*1.271*	*3.139*	0.003	60.2%	0.057
	Caucasian	5	*2.054*	*1.093*	*3.862*	*0.025*	82.4%	0.000
	Other	1	*1.310*	*1.034*	*1.660*	*0.025*	NA	NA
	Case control	1	1.720	0.862	3.433	0.124	NA	NA
	Cohort	2	1.637	0.894	2.996	0.110	61.6%	0.107
	Cross-sectional	7	*1.980*	*1.312*	*2.987*	*0.001*	79.6%	0.000
ODI	Total	5	*1.716*	*1.325*	*2.221*	*4.13 × 10*^*−5*^	36.8%	0.176
Sleep duration(≤ 5 h)	Total	19	*1.448*	*1.252*	*1.674*	*5.73 × 10*^*−7*^	90.2%	0.000
	African	1	*3.480*	*1.692*	*7.158*	*0.001*	NA	NA
	Asian	8	*1.219*	*1.002*	*1.484*	*0.048*	64.1%	0.050
	Caucasian	10	*1.589*	*1.396*	*1.808*	*2.38 × 10*^*−12*^	77.1%	0.012
	Cross-sectional	10	*1.692*	*1.475*	*1.941*	*6.38 × 10*^*−14*^	74.5%	0.000
	Cohort	9	1.218	0.995	1.491	0.055	77.0%	0.000
Sleep duration(≤ 6 h)	Total	18	*1.138*	*1.036*	*1.250*	*0.006*	53.6%	0.000
	Asian	8	1.127	0.963	1.319	0.136	50.6%	0.048
	Caucasian	9	*1.181*	*1.050*	*1.329*	*0.006*	39.3%	0.000
	Other	1	0.930	0.660	1.310	0.678	NA	NA
	Cross-sectional	9	*1.190*	*1.047*	*1.353*	*0.008*	61.3%	0.008
	Cohort	9	1.077	0.941	1.231	0.282	23.2%	0.237
Sleep duration(≤ 7 h)	Total	13	*1.196*	*1.064*	*1.344*	*0.003*	83.4%	0.000
	Asian	9	*1.081*	*1.016*	*1.150*	*0.014*	37.0%	0.123
	Caucasian	3	1.438	0.863	2.396	0.164	90.8%	0.000
	Other	1	*2.200*	*1.503*	*3.220*	*4.91 × 10*^*−5*^	NA	NA
	Cross-sectional	10	*1.207*	*1.051*	*1.387*	*0.008*	87.4%	0.000
	Cohort	3	*1.174*	*1.009*	*1.368*	*0.038*	0.0%	0.838
Sleep duration(≥ 8 h)	Total	15	*1.129*	*1.033*	*1.235*	*0.008*	54.3%	0.006
	Asian	9	*1.176*	*1.020*	*1.356*	*0.016*	57.7%	0.015
	Caucasian	6	1.086	0.931	1.266	0.258	41.2%	0.131
	Cross-sectional	11	*1.148*	*1.040*	*1.267*	*0.006*	62.0%	0.003
	Cohort	4	1.025	0.793	1.326	0.849	30.0%	0.232
Sleep duration(≥ 9 h)	Total	9	*1.162*	*1.057*	*1.279*	*0.002*	39.8%	0.102
	Asian	2	*1.181*	*1.079*	*1.292*	*3.06 × 10*^*−4*^	0.0%	0.646
	Caucasian	7	1.094	0.884	1.354	0.410	54.1%	0.042
	Cross-sectional	7	*1.158*	*1.034*	*1.296*	*0.011*	50.0%	0.062
	Cohort	2	1.147	0.841	1.565	0.385	21.9%	0.258
Sleep duration(≥ 10 h)	Total	6	*1.411*	*1.066*	*1.866*	*0.016*	90.4%	0.000
	Asian	1	*1.520*	*1.253*	*1.844*	*2.14 × 10*^*−5*^	NA	NA
	Caucasian	5	1.359	0.942	1.960	0.101	92.2%	0.000
	Cross-sectional	1	*1.410*	*1.332*	*1.492*	*1.91 × 10*^*−32*^	NA	NA
	Cohort	5	1.341	0.826	2.175	0.235	92.3%	0.000
Sleep quality	Total	11	*1.380*	*1.082*	*1.760*	*0.009*	96.0%	0.000
	Asian	10	*1.347*	*1.046*	*1.736*	*0.021*	96.3%	0.000
	Caucasian	1	*1.840*	*1.133*	*2.988*	*0.014*	NA	NA
	Cross-sectional	9	*1.514*	*1.160*	*1.977*	*0.002*	96.7%	0.000
	Case control	2	0.819	0.575	1.166	0.267	0.0%	0.839
Snoring	Total	9	*1.939*	*1.410*	*2.665*	*4.50 × 10*^*−5*^	92.9%	0.000
	Asian	4	1.665	0.885	3.133	0.114	93.1%	0.597
	Caucasian	5	*2.186*	*1.578*	*3.029*	*2.48 × 10*^*−6*^	78.9%	0.520
	Cross-sectional	6	*1.917*	*1.106*	*3.323*	*0.020*	91.4%	0.000
	Cohort	3	*2.051*	*1.402*	*3.001*	*2.16 × 10*^*−4*^	86.8%	0.000

the italic numbers means the OR values are statistically significant

### Oxygen desaturation index and hypertension

As shown in Table 1, five datasets from four articles were considered in analysis. Significant results were found for the association between 3% oxygen desaturation index and hypertension (OR = 1.72, 95%CI 1.33–2.22).

### Short sleep duration and hypertension

Twenty-nine articles were recruited in meta-analysis, including 19 datasets for sleep duration less than or equal to 5 h, 18 datasets for sleep duration less than or equal to 6 h, and 13 datasets for sleep duration less than or equal to 7 h. Sleep duration less than or equal to 5, 6, and 7 h was associated with hypertension, while in subgroup analysis, for sleep duration less than or equal to 5 h, the significant result was not found in cohort studies (OR = 1.218, 95%CI 0.955–1.491). In cross-sectional studies, sleep duration less than or equal to 5, 6, and 7 h was associated with hypertension (Table 1).

### Long sleep duration and hypertension

Fifteen articles were included in meta-analysis, in which, 15 datasets were for sleep duration more than or equal to 8 h, nine datasets for sleep duration more than or equal to 9 h, and five datasets for sleep duration more than or equal to 10 h.

Sleep duration more than or equal 8 h was associated with hypertension in whole population, while in cohort studies, significant association was not observed (OR = 1.025, 95%CI 0.793–1.326). Sleep duration more than or equal to 9 h was a risk factor of hypertension (OR = 1.162, 95%CI 1.057–1.279), same result was found for sleep duration more than or equal to 10 h; however, the heterogeneity was very high for this analysis (*I*^*2*^ = 90.4%).

### Snoring and hypertension

Five articles were eligible for meta-analysis. In total population, snoring was found to be risk factor (OR = 1.939, 95%CI 1.410–2.665). Same results were found in cohort studies and in all ethnic subgroups.

### Sleep quality and hypertension

Seven articles reported the association between sleep quality and hypertension. In total population, the significant association was found (OR = 1.380, 95%CI 1.082–1.760). No significant result was found in case-control studies (OR = 0.819, 95%CI 0.575–1.166).

### Heterogeneity, sensitivity analysis, and bias

As shown in Table 1, the heterogeneity was varied for different factors. Very high heterogeneity was observed in analyses for association of sleep quality, snoring, and sleep duration more than or equal to 10 h. High heterogeneity was found in these meta-analyses. Meta-regression analysis and subgroup analysis were conducted in this study, and high heterogeneity was improved in some subgroups (see supplementary 3). We performed sensitivity analysis to evaluate the stability of results and found that the summary ORs did not change, except for the association of sleep duration more than or equal to 10 h and hypertension. Egger and Begg’s tests were conducted to evaluate the publication and small study bias; publication bias and small study bias were found in the association of OSA and hypertension (*P* = 0.04 and 0.01, respectively).

## Discussion

Hypertension is a major risk factor for cardiovascular diseases. In addition to that, its complications also include heart failure, peripheral vascular disease, renal impairment, retinal hemorrhage, and visual impairment [17]. This meta-analysis was conducted to thoroughly explore the association between sleep status and hypertension. In this study, a total of 54 studies involving 1,074,207 subjects were included, and 114 datasets were analyzed. Six types of sleep status were assessed. The results indicated that OSA, ODI, sleep quality, short or long sleep duration, and snoring were the risk factors of hypertension. However, the association was varied in different subgroups. The publication bias was only found in meta-analysis for association between OSA and hypertension. The sensitivity analysis indicated that the pooled results were stable.

In terms of OSA, positive results were found in all study design groups. For short sleep duration less than or equal to 5 h, it is a risk factor for hypertension in the whole population, which is consistent with previous findings [8]. Notably, in Asian population, the significant result was not observed in analysis; we found there were two studies in China with large sample sizes reported negative results [10, 18]. Long sleep duration is also considered a factor of hypertension, for sleep duration more than or equal to 8 h, there is no significant result found except in Asian population. However, chronic long sleep duration became a risk factor in whole population when the number of hours is more than 9 h in adults. Among seven studies regarding snoring, several studies reported that snoring people had a higher risk of hypertension [19–21]. However, some other researchers found that there was no significant association between snoring and hypertension [22]. This study found snoring is an important factor of hypertension with an odds ratio of 1.38.

The association between sleep status and hypertension may be explained by some potential mechanisms. For OSA, the blood pressure may be attributed to OSA-induced sympathetic activation, which heightens vascular resistance and cardiac output by stimulating the rennin-angiotensin-aldosterone system [6]. For short sleep duration, some experimental studies have reported that increased blood pressure may be associated with heightened sympathetic nervous system activity after nights. It is also found that short sleep duration can disrupt circadian rhythmicity and autonomic balance [23]. Another hypothesis indicates that depriving sleep hours is a stressful condition, which has been proved to promote salt appetite and suppress renal salt fluid excretion [24], excessive salt intake is confirmed as a risk factor of hypertension. This study searched articles on the relationship between sleep status and hypertension. Then, we conducted the meta-analyses to comprehensively assess the association between them. Even though the results were credible, there were still some limitations. First of all, there are several analyses with high heterogeneity, and it affects the reliability of these results. Secondly, we found the publication bias and small study bias in analysis of association between OSA and hypertension; thus, our results need to be confirmed by more high-quality literatures. Even though these limitations existed, our findings are still valuable because of the large sample size, the comprehensive factors, and the credible analytical methods.

In conclusion, the hypertension risk might be reduced if patients with OSA, ODI could be treated. Chronic short sleep duration or long sleep duration are harmful for hypertension prevention. Moreover, treating snoring is an effective measure to prevent hypertension. However, our findings should be supported by more studies with large sample sizes and high qualities.

## Electronic supplementary material

ESM 1 (RAR 445 kb)
